# Life time suicidal thoughts in an urban community in Hanoi, Vietnam

**DOI:** 10.1186/1471-2458-6-76

**Published:** 2006-03-26

**Authors:** Huong Tran Thi Thanh, Trung Nam Tran, Guo-Xin Jiang, Antoon Leenaars, Danuta Wasserman

**Affiliations:** 1 Hanoi Medical University, Hanoi, Vietnam; 2 Swedish National and Stockholm County Centre for Suicide Research and Prevention of Mental Ill-Health (NASP) at the National Institute of Psychosocial Medicine and Department of Public Health Sciences, Karolinska Institute, Stockholm, Sweden; 3 Department of Medical Epidemiology and Biostatistics, Karolinska Institute, Stockholm, Sweden; 4 Principal investigator

## Abstract

**Background:**

Suicidal thought is a risk factor and a stage in the suicidal process from planning to attempting and dying by suicide. To date, studies on suicidal thought in the general population, especially in Asian communities, have been limited.

**Method:**

The WHO SUPRE-MISS (the multisite intervention study on suicidal behaviours) community survey questionnaire was filled in for 2,280 randomly selected residents of the DongDa district of Hanoi, Vietnam by means of face-to-face interviews. This multi-factor questionnaire includes such variables as sociodemographic information, suicidal thought and history of suicide attempts, physical health, alcohol consumption and medication.

**Results:**

Prevalence rates for life time suicidal thoughts, suicide plans and suicide attempts were 8.9%, 1.1% and 0.4% respectively. Suicidal thoughts are associated with multiple characteristics, such as female gender, single/widowed/separated/divorced marital status, low income, lifestyle (use of alcohol, sedatives and pain relief medication), but not with low education or employment status. Having no religion and being a Buddhist appear to be protective factors for suicidal thought.

The ratio of suicidal thoughts, suicide plans and suicide attempts on a lifetime basis is 22.3:2.8:1.

**Conclusion:**

In Vietnam, as in Western and other Asian countries, suicidal thoughts are associated with similar negative psychosocial risk factors, lifestyle and emotional problems, which implies that suicide preventive measure developed elsewhere can be adjusted to Vietnamese condition. Understanding the unique and common risks in a culture may assist in prediction and control.

## Background

Suicide is an international public-health problem. It is now among the five leading causes of death among young people worldwide. Although the highest suicide rates are currently found in Eastern Europe, the largest number of suicides takes place in Asia. Suicidal behaviour is often a process: from death wishes to suicidal thoughts, suicide attempts. [1]. This process has been known for decades [2], but predicting which individuals will proceed from thoughts to death by suicide is poorly understood. Nonetheless, suicidal thought has been shown to be an important short-term risk factor for death by suicide [3,4] However, since such thoughts are not rare, identifying persons who wish committed suicide among the people with suicidal thoughts is difficult, but also critical to prediction and control [5].

Several investigators in the West have found sociodemographic and health-related factors associated with suicidal thought: such as advanced age, female sex, financial problems and somatic and mental diseases [6-13]. However, limited studies on suicidal thought have been carried out among the general population, especially in Asian communities.

This study is a part of the World Health Organization (WHO) SUPRE-MISS (multisite intervention study on suicidal behaviours) study, which is being undertaken to understand suicide better in neglected and culturally diverse regions, such as China, India, South Africa and Vietnam [14]. This is a global step forward, based on the WHO/Euro Multicentre Study on Suicidal Behaviour [15]. These extensive studies are the best-known empirical studies on suicide risk, with implications for prevention. The WHO SUPRE-MISS is a natural next step that forms part of these culturally diverse investigations [14].

Suicide was among the 10 leading causes of death in Vietnam in 2002, and the estimated suicide rate is 0.98/100,000 according to the Ministry of Health based on the hospital records of persons who died. [16] Our previous study on suicide attempters in Vietnam [17], showed the key role of psychosocial stressors in attempted suicide, but to date there has been no study on suicide ideators.

The purpose of the study was to investigate the frequency and characteristics of persons with lifetime suicidal thoughts in the general population in an urban community in Vietnam. Specific characteristics studied included sociodemographic factors, physical health and lifestyle factors, such as the use of alcohol and sedatives. The aim was to isolate the factors and characteristics that may have a critical bearing on risk prediction among suicide ideators in Vietnam and allow future geographical and cultural comparisons [14].

## Methods

### Data collection

From March to May 2003, we conducted a questionnaire survey among residents of Dong Da, the largest and most populated district of Hanoi, Vietnam. A simple random sample (a sampling procedure that ensures that each person in the population has a chance of being selected), using a table of random numbers, yielded 2,400 subjects that were selected from the list of district residents (numbering some 350,000). This list was compiled from the lists from all the municipalities in the Dong Da district, provided by the respective municipal People's Committees. No exclusion criteria were applied, except that the subjects were aged 14 or more. There are 4 persons who were treated in out-patient departments for mental disorders were also excluded from the study. The selected subjects were contacted by the staff of the municipal People Committee to make the appointment for the interviewers' visits. At each visit to the subject's home, the interviewer approached the potential participant, briefly described the study and requested their participation. A total of 2,280 subjects agreed to take part, resulting in a participation rate of more than 94%. 2,260 participants who responded to questions about suicidal thoughts and suicide attempts are included in the analyses. General characteristics of the study material are presented in the results.

### Instrument

Participants were interviewed face to face, using the SUPRE-MISS community survey questionnaire [14], translated into Vietnamese and adapted by a group composed of psychiatrists, psychologists and professionals in public-health sciences at Hanoi Medical University and the National Institute for Mental Health in Vietnam. The questions elicited detailed sociodemographic background information and explored respondents' suicide-related behavior, physical health, emotional problems and use of drugs and alcohol.

The questions concerning suicidality were as follows, for the respondent's lifetime and the last 12 months:

• "Have you ever seriously thought of committing suicide?"

• "Have you ever made a plan to commit suicide?"

• "Have you ever attempted suicide?"

The questions concerning to emotional problems was as follow:

• "Do you or did you ever experience for prolonged periods of time (for over at least one year) troubles within yourself that hindered your functioning? (e.g.: fears of places, anxiety, depressive feeling, hopelessness...)

The DRUG CARD is used for getting information on habit using alcohol, sleeping pills, pain relief medication in life time [14]. The DRUG CARD includes tobaco products, alcoholic beverages, marijuana, cocain, stimulants, inhalants, sleeping pills, hallucinogens, heroin, pain medication.... and the question concerning to the habit using these kind of drug was as follow:

• " In your life, which of the following substances (see DRUG CARD) have you ever used?"

### Interviews

A group of eight students in their final year of studying public health at Hanoi Medical University were carefully trained in the use of the questionnaire and interview techniques. The interviewers had a regular debriefing and training meeting throughout the study.

### Quality and reliability control

The interview process was monitored, checked and retested. Approximately 10% (200 persons) of the 2,280 subjects were selected randomly for reliability tests. Interviews were performed by telephone and sometimes repeated (test/retest). All the variables were tested and results were the same.

### Ethical approval

Participation in the study was anonymous and voluntary. Ethical approval was obtained from the Ministry of Health in Vietnam, Hanoi Medical University and the Ethical Committee at Karolinska Institute in Stockholm, Sweden. Each participant signed a consent form agreeing to take part in the study.

### Statistical analyses

Data were entered by two different persons using SPSS version 10 (SPSS Inc., Chicago, IL) and analysed using Stata version 8 (Stata Corp, College Station, TX). General characteristics of the study material were analysed by descriptive analyses. Comparisons of proportions between groups were conducted using χ^2 ^tests. Income was categorized into two levels, high and low, based on the median. Since the numbers of "widowed" and "divorced/separated" people were small, we combined these categories with "single" to make an "Unmarried" category. Similarly, for the subjects' religion, we combined "Protestant", "Jewish", "Muslim", "Greek Orthodox" and "Other" in the "Others" category. Multivariate logistic regression was used to examine the associations of independent variables with suicidal thoughts, simultaneously adjusting for potential confounders. Variable selection in the multivariate model was based on results presented in previous studies [18,19] and the most fitted model was selected.

## Results

### * General characteristics

Table 1 presents the general characteristics of the study sample. Of the 2,260 persons, 1167 (51.6%) were female and 1093 (48.4%) male. The mean and median ages of subjects were 40 and 37, respectively, and the age range was 14–96. The majority of subjects were married and/or living with a permanent partner (63%) or single (34%). Seventy per cent of the participants had attended upper secondary school or higher education. The mean and median monthly income were 500,000 and 600,000 Vietnam Dong (approx. USD 32 and 38.2) respectively. Most participants (91%) followed no religion. However, sixty five percent acknowledged a religious belief. Buddhists, Catholics and "Others" accounted for 5.6%, 1.8% and 1.5%, respectively. More than half (50.7%) were employed, whereas 21.3% and 17.1% were retired and studying respectively. Only 11% were unemployed or doing housework. As expected, females were relatively likely to be home-workers (15% vs. 4%) while males were more likely to be employed (55% vs. 46%) (P < 0.001). Males also had higher education than females (P = 0.01). Amongst the respondents 53.7% have ever used alcohol in their lifetime, whereas 10.4% and 9.4% have ever used sedatives and pain relief medication, respectively.

### * Prevalence of past suicidal thoughts

Of the 2,260 participants, 201 (8.9%) had thought about committing suicide at one time or another, and 74 (3.3%) in the past 12 months (Table 2). Among those 201 persons who had had suicidal thoughts, 25 persons (12.4%) had planned suicide at some stage. Among those who had planned suicide (n = 25), nine had attempted it, including three in the past 12 months. Eight respondents were aged below 25 at the time of their first suicide attempt. Regarding method, six people had used sedatives, while one had used alcohol and one a pesticide. For one person information was lacking about the method when attempting suicide.

The incidence of suicidal thoughts, suicide plans and suicide attempts during a lifetime was 22.3:2.8:1 (201:25:9 in Table 2). For the past 12 months, this ratio was 24.7:4.0:1 (74:12:3 in Table 2)

### * Suicidal thoughts and physical illness or symptoms

Twelve of the 201 (6%) people with suicidal thoughts had had chronic joint pain for at least a year. Members of the group with suicidal thoughts had also suffered from headache (4 persons), cancer (3), stomach ache (3), asthma (2), and hepatitis (2). In the group without suicidal thoughts, 71 had chronic joint pain, 22 headaches, one cancer, 34 stomach aches, two asthma and three hepatitis. Although the proportion of respondents with physical illnesses in the "suicidal thought group" was often higher than that of the "non-suicidal thought groups", these differences are not statistically significant, except for asthma (P < 0.05) and cancer (P < 0.01).

### * Suicidal thoughts and feelings of anxiety, depression and fear for at least one year

In the group who had suicidal thoughts, there were 13 (6.5%) persons with feelings of anxiety, 12 (6.0%) persons who had experienced depression and 6 (3.0%) participants who had felt fear for at least one year. The number of respondents with non-suicidal thoughts was 20 (1.0%) with anxiety, 17 (0.8%) with depression and 2 (0.1%) with fear. The difference between two groups with respect to each kind of feeling is statistically significant (p < 0.01).

### * Correlates of suicidal thoughts

Table 3 presents the results of the univariate for correlates of various factors with lifetime suicidal thoughts. Youth, being female, being unmarried (single, widowed, divorced or separated), being a homeworker or a student compared with being employed; low income; and use of sedatives and analgesics were associated with lifetime suicidal thoughts. In multivariate analysis (table 3), we simultaneously adjusted for age, gender, marital status, education, employment status, religion, and use of alcohol, sedatives and analgesics. In accordance with univariate analysis, the factors of being female (OR = 2.5; 95% CIs 1.7–3.7) and unmarried (OR = 3.3; 95% CIs 2.1–5.4), having a low income (OR = 1.7; 95% CIs 1.1–2.6) and having taken sedatives (OR = 2.7; 95% CIs 1.7–4.3), and analgesics at one time or another (OR = 2.6; 95% CIs 1.6–4.1) are independently associated with having suicidal thoughts. In contrast with univariate analysis, use of alcohol was also found to be associated with suicidal thoughts (OR = 1.6; 95% CIs 1.1–2.2). Although not statistically significant at the 0.05 level, followers of Muslim, Jewish, Protestant and Greek Orthodox religions were more likely have had suicidal thoughts than those who followed no religion (OR = 2.7; 95% CIs 0.9–7.3). Low education and the occupations of homeworker and student were not found to be associated with suicidal thought.

## Discussion

Information on mental health, especially suicidal behaviours, is lacking in Vietnam: this is the first study on suicidal thought in a Vietnamese general population.

### Methodology

In this investigation, face-to-face interviews were conducted. This procedure not only allows any misunderstanding of the questions to be clarified, but also helps to prevent non-participation. Some previous studies using questionnaires sent by post or administered by telephone have shown higher participation refusal rates than in this study [20,21]. Even though the analysis of the data was anonymous to the researchers, and thus de-identified, the participants were not anonymous during the sampling prosedure. The answeres given might differ from data given in a totally anonymous situation. The procedure of training, pilot study, monitoring, checking, and retesting was conducive to inter-interviewer reliability.

However, the perception by the interviewed person that the interviewers were the part of the Commune' People Committee and the role of stigma cannot be ruled out. The rate of suicidal thoughts reported in the study was nearly 8.9%, but the real proportion may be higher for several conceivable reasons. People cannot remember suicidal thoughts throughout their lives, especially if no particular events take place to remind them of these thoughts. Beyond issues of memory, cultural aspects may have affected people's willingness to inform others that they have had suicidal thoughts or attempted suicide in their lifetime. This factor is assumed to be prevalent in Asian countries [22,23].

The procedure of training, pilot study, and re-testing assured the inter-interviewer reliability. Understanding the meaning of questions on suicidal thoughts can also cause the problem of the reliability of the obtained results. A particularly low rate of lifetime suicide attempts (0.4%) calls for further investigation. The problem of bias due to social desirability of respondents is a well known phenomenon in this kind of studies [24].

The study design is a cross-section survey, so no conclusions could be drawn concerning the causality between suicidal thinking and other factors. Nonetheless, it helps to indicate a direction for further longitudinal studies.

### Suicidal thoughts, suicide plans and suicide attempts during lifetime and past 12 months

Lifetime prevalence for suicidal thoughts varies among countries, and ranges from 2.1% to 18.5% in studies of nine countries [23]. The rates of suicidal thoughts on a lifetime basis and/or in the past twelve months are lower in our study than in studies in Western countries [23,25] and China but higher than in India [14]. This may be due to cultural differences but also, in part, to the methodology used. In the nine-country study [23], for example, the Diagnostic Interview Schedule, Version III and the DSM-III were used and suicidal thought was assessed by the question" Did you ever feel so low you thought of committing suicide?" which differs from the questions about suicidal thoughts used in this study [14]. The prevalence of suicide attempts in Vietnam was the lowest among countries within the SUPRE-MISS framework, when the same methodology was used [14]. But, Vietnam reported more serious suicide attempts, based on the self-evaluation of seriousness in comparison with answers from the general population in other countries [14]. Real suicide rate in Vietnam is unknown because the rate reported by the Ministry of Health is based only on hospital data. However, most of suicides occur outside the hospital systems. There is no national system to monitor causes of death, including suicide in Vietnam. Therefore, surveillance and registration of suicide and other causes of death should be developed.

The ratio of suicidal thoughts, suicide plans, and suicide attempts in Vietnam was 22.3:2.8:1 on a lifetime basis. Suicidal thoughts are 7 times more prevalent than suicide plans, while the latter are only approximately three times as common as suicide attempts. There is the same ratio between suicide plans and suicide attempts in Vietnam (2.8:1) as in China, Australia, Stockholm but different from India, Brazil, Sri Lanka, Iran and Estonia where ratio between suicide plans and suicide attempts is in the range between 1:0.7 to 1:1.7 [14]. The ratio between suicidal thought and suicide attempts in Vietnam is 22.3: 1 which differs from Western and other Asian countries, where the ratio between suicidal thoughts and suicide attempts is smaller. A future in-depth study will be undertaken to understand the reasons for the differences.

### Correlates of having suicidal thoughts

In our urban Vietnam sample, female gender is strongly associated with suicidal thoughts compared with male. This result is consistent with most of previous studies [25]. In contrast to our finding, the Finnish study reported a higher prevalence of suicidal thoughts in men than in women [26].

Suicidal thoughts are associated with single marital status or life events such as divorce, widowhood and separation. Marriage seems to be a protective factor for suicidal thoughts. These factors have, in fact, been identified in various studies in Asian and Western countries alike [22,27-29].

In our study, the results show that participants who followed the Buddhist religion experienced less suicidal thought than those who either had no religion or followed other religions (Muslim, Protestant, Jewish). Most Vietnamese people are strongly influenced by Buddhist practices, even if they do not openly admit that they are Buddhists [30]. This cultural and religious tradition may possibly explain the low level of suicidal thoughts and suicide attempts in Vietnam compared with other countries [14,23].

Suicidal thoughts in this study were frequent in young adults. The results of our previous study on hospitalised suicide attempters [17] showed that the largest group of patients was the group aged 15–24 years.

Several previous studies found the correlation between low income, unemployment with suicidal thought [27,31]. It is similar to the results in this study, which show that there are only 1.4% of respondents who are unemployment but there are 44.4% who report that they have a low level of income. The unsolved financial problems for a long time can lead to distress and depression symptoms. Poor economy influences also the access to the health care service in the developing countries as Vietnam.

### Suicidal thoughts and mental problems, use of alcohol, sedatives and pain relief medication

The result of our study show that nearly 90% of respondents never used sedatives or pain relief medication in their life. The habit of using this kind of medication might be a protective factor for suicidal thought in Vietnam.

Prolonged feelings of anxiety, depression and fear are predictors for suicidal thought. This research calls for training of primary health workers, and this is consistent with the WHO strategies for suicide prevention [32]. It is well known that many people who suffer from stress and mental problems over long periods do not come to the health services' attention [7] This may be connected with taboos or diverse cultural aspects of mental illness in Vietnam, but also due to a general lack of knowledge about diagnostics and treatment of mental illness [33].

Suicidal thinking is related to the use of sedatives and these kinds of medicines are a frequent means of attempting suicide in Vietnam [17]. Public-health approaches that call, for example, for careful management of medication of this kind or selling smaller pack sizes are suggested as effective means of preventing suicidal behavior [34].

Suicidal thought is highly correlated with use of alcohol on a lifetime basis. Studies from non-Asian countries illustrated the same findings [10,35]. But in our previous study, alcoholism and alcohol addiction were rarely found in hospitalized suicide attempters [17] which may be due to the lack of attention by doctors to diagnose alcohol misuse and abuse.

### Suicidal thoughts and physical health

Suicidal thoughts and attempted suicide are related not only to depression and other mental disorders, but also to general medical illnesses ranging from terminal diseases such as cancer and AIDS to acute life-threatening illnesses such as stroke, traumatic brain injury and spinal cord injury, and also more widespread illnesses, such as asthma and chronic bronchitis [9]. Although the numbers of cancer and asthma patients in our study were small as they were not selected from the hospital, suicidal thoughts were found to be significantly more frequent in these groups. The results indicate the need of paying attention to suicidal thoughts when treating somatically ill patients and the need for further studies focusing on hospitals.

## Conclusion

This study isolated risk factors for suicidal thought and thus the suicidal process – in the DongDa district of Hanoi, Vietnam. Many of these factors are similar to Western and other Asian countries, which has the implication that similar preventive and treatment measures can probably be used.

Suicidal thoughts are multi-determined, associated with mental problems, such as feelings of anxiety and depression; general medical illness, such as cancer; and an array of social factors. Understanding better the unique and common complex factors in a culture or region may facilitate prediction and control of the suicidal process in the region concerned.

## Competing interests

The author(s) declare that they have no competing interests.

## Authors' contributions

DW and HTTTdevised the idea of the study. HTTT coordinated the surveys. The analyses of data were carried out by TNT and HTTT. HTTT produced the first draft and all authors revised and contributed to the final version of the manuscript.

## Pre-publication history

The pre-publication history for this paper can be accessed here:



## Figures and Tables

**Table 1:** Characteristics of the study sample

	**Characteristics**	**N**	**%**
**Age (in years)**	<25	556	24.9
	25 – 34	491	21.9
	35 – 44	351	15.7
	45 – 54	332	14.8
	55 – 64	248	11.1
	≥65	260	11.6
**Gender**	Male	1093	48.4
	Female	1167	51.6
**Marital status**	Single	767	34.0
	Married/permanent partners	1425	63.1
	Widowed	43	1.9
	Divorced/separated	22	1.0
**Religion**	Buddhist	127	5.6
	Christian	40	1.8
	Others (Muslim, Protestant, Jewish)	35	1.5
	No religion	2055	91.1
**Religiousness**	Yes	1367	65.4
	No	722	34.6
**Education**	None/primary	204	9.1
	Secondary	470	20.8
	Upper secondary but no higher	1125	49.9
	Higher	405	18.0
	Postgraduate	50	2.2
**Income**	Low	1161	44.4
	High	932	55.6
**Employment**	Employed	1144	50.7
	Unemployed	32	1.4
	Retired	480	21.3
	Housework	214	9.5
	Student	387	17.1
**Ever used alcohol**	Yes	1212	53.7
	No	1045	46.3
**Ever used sedatives**	Yes	235	10.4
	No	2017	89.6
**Ever used pain relief medication**	Yes	212	9.4
	No	2041	90.4

**Table 2:** Suicidal thoughts, suicidal plans, and suicide attempts during life-time and in the past 12 months in the general population (n = 2260)

	**Male **n (%)	**Female **n (%)	**Total **n (%)
**Thought about suicide**			
During lifetime	61 (5.6)	140 (11.9)	201 (8.9)
In the past 12 months	21 (1.9)	53 (4.5)	74 (3.3)
**Planned suicide**			
During lifetime	11 (1.0)	14 (1.2)	25 (1.1)
In the past 12 months	4 (0.4)	8 (0.7)	12 (0.5)
**Attempted suicide **			
During lifetime	5 (0.4)	4 (0.3)	9 (0.4)
In the past 12 months	2 (0.2)	1 (0.1)	3 (0.1)

**Table 3:** Risk factors for life time suicidal thoughts

			**Univariate analysis**		Multivariate analysis* (N = 1916)	
		**Suicidal thought n (%)**	**Odd Ratios**	**95% CIs**	**Adjusted OR**	**95%CIs**

**Age **(in years) N = 2238	< 25	76 (13.7)	3.3	1.7–6.1		
	25 – 34	44 (9.0)	2.0	1.1–3.9		
	35 – 44	20 (5.7)	1.2	0.6–2.6		
	45 – 54	32 (9.6)	2.2	1.1–4.4		
	55 – 64	15 (6.0)	1.3	0.6–2.9		
	≥65	12 (4.6)	1	-		
**Gender **N = 2260	Female	140 (12.0)	2.3	1.7–3.1	2.5	1.7–3.7
	Male	61 (5.6)	1	-	1	-
**Marital status **N = 2257	Unmarried	114 (13.7)	2.4	1.8–3.3	3.3	2.1–5.4
	Married	87 (6.1)	1	-	1	-
**Religion **N = 2257	Buddhism	9 (7.1)	0.8	0.4–1.6	0.5	0.2–1.2
	Catholic	4 (10)	1.1	0.4–3.2	1.1	0.4–3.5
	Others	5 (14.3)	1.7	0.6–4.5	2.7	0.9–7.6
	No religion	182 (8.9)	1	-	1	-
**Religiouness **N = 2089	Yes	88 (44.9)	1.6	1.1–2.2		
	No	634 (33.5)	1	-		
**Employment status **N = 2257	Unemployed	3 (9.4)	1.2	0.4–4.2		
	Retired	29 (6.0)	0.8	0.5–1.2		
	Housework	26 (12.1)	1.7	1.1–2.7		
	Student	56 (14.5)	2.0	1.4–2.9		
	Employed	87 (7.6)	1	-		
**Income **N = 2093	Low	131 (11.3)	1.9	1.4–2.7	1.7	1.1–2.6
	High	57 (6.1)	1	-	1	-
**Education **N = 2254	Secondary school or lower	58 (8.6)	1.1	0.8–1.4		
	Upper secondary school or higher	143 (9.0)	1			
**Ever used alcohol? **N = 2257	Yes	118 (9.7)	1.3	0.9–1.7	1.6	1.1–2.2
	No	83 (7.9)	1	-	1	-
**Ever used sedatives? **N = 2252	Yes	44 (18.7)	2.7	1.9–4.0	2.7	1.7–4.3
	No	156 (7.7)	1	-	1	-
**Ever used pain relief medication? **N = 2253	Yes	39 (18.4)	2.6	1.8–3.9	2.6	1.6–4.1
	No	161 (7.9)	1	-	1	-

* The model included age, gender, marital status, education level, income, religion, religiousness, employment status, use of alcohol, sedatives, and pain relief medication. Only variables with P-value <0.1 are presented.
